# Supplementary material for: Causal Relationship between Aging and Anorexia Nervosa: A White-Matter-Microstructure-Mediated Mendelian Randomization Analysis
Source: Biomedicines. 2024 Aug 16;12(8):1874. doi: 10.3390/biomedicines12081874 (PMC11351342; doi:10.3390/biomedicines12081874)
Supplement: Supplementary file 1 [file biomedicines-12-01874-s001.zip › Supplementary Figure/Figure S4/Singe SNP plot of MR analysis for TL to ubm-b-1553.pdf]

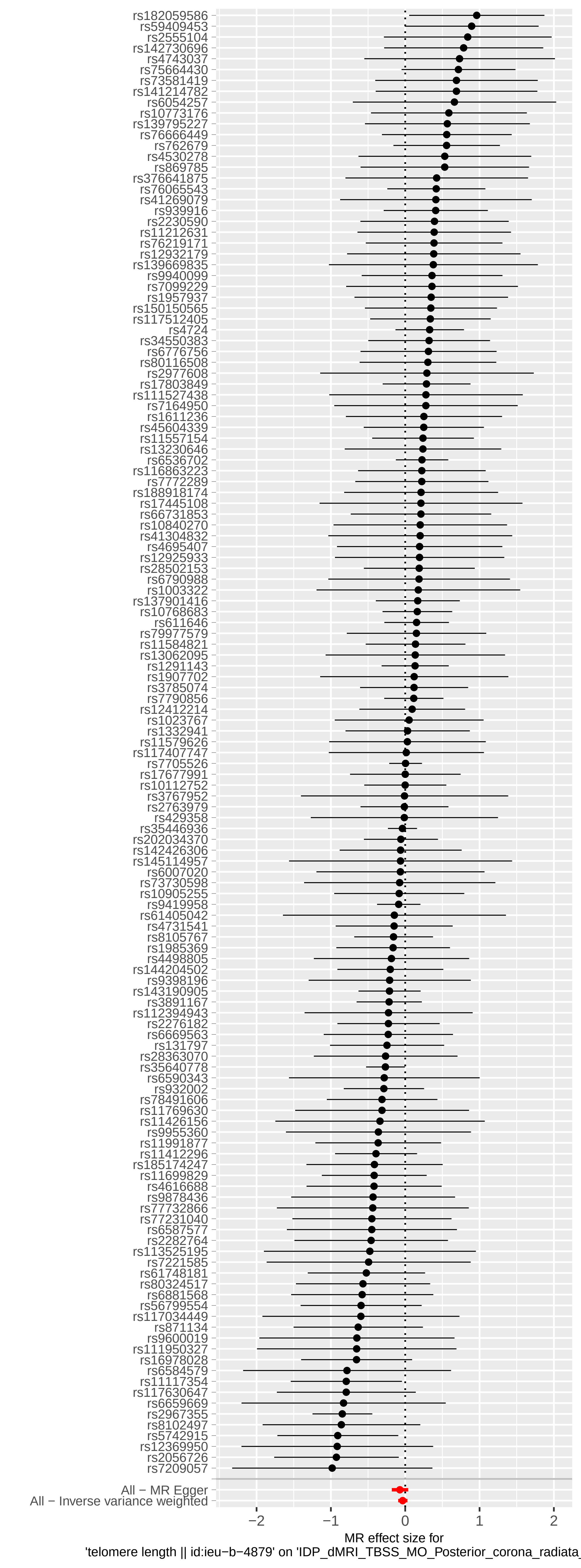
rs182059586
rs59409453
rs2555104
rs142730696
rs4743037
rs75664430
rs73581419
rs141214782
rs6054257
rs10773176
rs139795227
rs76666449
rs762679
rs4530278
rs869785
rs376641875
rs76065543
rs41269079
rs939916
rs2230590
rs11212631
rs76219171
rs12932179
rs139669835
rs9940099
rs7099229
rs1957937
rs150150565
rs117512405
rs4724
rs34550383
rs6776756
rs80116508
rs2977608
rs17803849
rs111527438
rs7164950
rs1611236
rs45604339
rs11557154
rs13230646
rs6536702
rs116863223
rs7772289
rs188918174
rs17445108
rs66731853
rs10840270
rs41304832
rs4695407
rs12925933
rs28502153
rs6790988
rs1003322
rs137901416
rs10768683
rs611646
rs79977579
rs11584821
rs13062095
rs1291143
rs1907702
rs3785074
rs7790856
rs12412214
rs1023767
rs1332941
rs11579626
rs117407747
rs7705526
rs17677991
rs10112752
rs3767952
rs2763979
rs429358
rs35446936
rs202034370
rs142426306
rs145114957
rs6007020
rs73730598
rs10905255
rs9419958
rs61405042
rs4731541
rs8105767
rs1985369
rs4498805
rs144204502
rs9398196
rs143190905
rs3891167
rs112394943
rs2276182
rs6669563
rs131797
rs28363070
rs35640778
rs6590343
rs932002
rs78491606
rs11769630
rs11426156
rs9955360
rs11991877
rs11412296
rs185174247
rs11699829
rs4616688
rs9878436
rs77732866
rs77231040
rs6587577
rs2282764
rs113525195
rs7221585
rs61748181
rs80324517
rs6881568
rs56799554
rs117034449
rs871134
rs9600019
rs111950327
rs16978028
rs6584579
rs11117354
rs117630647
rs6659669
rs2967355
rs8102497
rs5742915
rs12369950
rs2056726
rs7209057
All − MR Egger
All − Inverse variance weighted
−2
−1
0
1
2
MR effect size for
'telomere length || id:ieu−b−4879' on 'IDP_dMRI_TBSS_MO_Posterior_corona_radiata_
